# Sickly Sweet: Insecticidal Polyols Induce Lethal Regurgitation in Dipteran Pests

**DOI:** 10.3390/insects10020053

**Published:** 2019-02-12

**Authors:** Francisco Díaz-Fleischer, José Arredondo, Rodrigo Lasa, Carlos Bonilla, Diana Debernardi, Diana Pérez-Staples, Trevor Williams

**Affiliations:** 1 INBIOTECA, Universidad Veracruzana, Apartado Postal 250, Xalapa, Veracruz 91000, Mexico; bonillamanuel033@gmail.com (C.B.); dgdebernardih@hotmail.com (D.D.); diperez@uv.mx (D.P.-S.); 2 Programa Moscafrut SAGARPA-SENASICA, Camino a los Cacaotales S/N, Metapa de Domínguez, Chiapas CP 30860, Mexico; jose.arredondo@iica-moscafrut.org.mx; 3 Instituto de Ecología AC, Apartado Postal 63, Xalapa, Veracruz 91070, Mexico; rodrigo.lasa@inecol.mx

**Keywords:** propylene glycol, glycerol, toxicity, dipterans, feeding, regurgitation

## Abstract

Polyols are commonly used in food and medicines as sweeteners and preservatives but may also have insecticidal properties against some species of Diptera. Here we compared the insecticidal activity and feeding response of glycerol and propylene glycol (PG) on two tephritids: *Anastrepha ludens* and *Anastrepha obliqua*, and the drosophilid *Drosophila suzukii*. First, flies were exposed to solutions of 50% sucrose and the two polyols at concentrations of 1.67 M, 2.78 M and 4.18 M for 24 h and then observed at 24 h intervals for a period of three days. Both polyols elicited strong regurgitation behavior in the three flies and killed them. Regurgitation apparently also reduced flies’ body weight, and this was particularly apparent in insects that fed on 4.18 M PG solutions. A high percentage of individuals exposed to PG solutions perished after 72 h. The number of proboscis extensions, which is associated with feeding preference, was lower in the 4.18 M polyols + sucrose mixtures than in the 50% sucrose solution. Glycerol had a lower insecticidal effect in *Anastrepha* spp. and very little insecticidal effect in *D. suzukii*. Finally, elevated regurgitation and mortality was confirmed in *A. ludens* treated with 1.0–2.78 M of erythritol plus sucrose. Our results demonstrate that PG, and to a lower extent glycerol, have the potential for being used as a safer method of insect pest control. The hyper-regurgitation response may contribute to the insecticidal properties of these polyols in Diptera.

## 1. Introduction

Many species of Diptera are pests of agriculture and human and animal health through direct damage to products or individuals, or as vectors of parasites and pathogens. These insects are usually controlled using broad-spectrum chemical insecticides. If used incorrectly, these products can adversely affect farmer health and impact populations of non-target arthropods, including natural enemies and pollinators [1,2]. In the search for novel methods of pest control, researchers have focused on insect metabolism or specific organs or systems [3,4], such as the gut [5] or the excretory system [6], as targets for effective and selective insecticidal compounds.

The insect crop has been considered as a potential target for novel methods of pest control [7]. This organ comprises a muscular system of pumps and sphincters located in the initial section of the digestive system, where food storage and regurgitation are regulated [8]. Adult dipterans that feed on liquid meals often exhibit regurgitation, the function of which is to reduce the amount of water in food and thereby increase the nutritional value of each meal, but also to lower body temperature [7,9].

In the search for biorational methods for pest control with reduced risks for non-target organisms, recent studies have identified a selection of polyols (sugar alcohols) with insecticidal properties in some dipteran species [10,11,12,13,14] and ants [15]. The non-nutritive polyol, erythritol, has attracted particular attention due to its insecticidal effects that have been tentatively attributed to starvation or lethal osmotic properties [11,12]. Erythritol and several other polyols are FDA approved sweeteners and are consumed on a regular basis by humans with no adverse effects [15]. This suggests that polyols could contribute to safe and effective pest control strategies with little if any risk to human health.

In this study we tested two polyols, glycerol and propylene glycol (PG), compounds that are widely approved as food additives and are used as drug solvents, humectant food additives, as a moisturizer in medicines, cosmetics, and tobacco products [16] and that are readily consumed by tephritid and drosophilid adults [17,18]. We present evidence that these two previously untested polyols, glycerol and PG, cause rapid mortality in three species of Diptera of major agricultural importance: *Anastrepha ludens* and *A. obliqua*, tephritid pests of citrus, mango and other fruits in the Americas [19] and *Drosophila suzukii*, an invasive pest of berries and soft-skinned fruit [20]. Moreover, we demonstrate that these polyols and another polyol sweetener, erythritol, exert their toxic effects by inducing a continuous regurgitation response that is rapidly lethal to these insects. Additionally, we examined the proboscis extension reflex (PER) to determine the likely feeding responses of these species to each of the polyols tested.

## 2. Materials and Methods

### 2.1. Insects and Compounds

*Anastrepha ludens* and *A. obliqua* flies were obtained as pupae produced at the Moscafrut Plant (SAGARPA-SENASICA) in Metapa de Domínguez, Chiapas, Mexico and were reared as described elsewhere [21]. Flies emerged in four different wooden-framed cages of 30 cm × 30 cm × 30 cm covered with nylon mesh. After emergence, insects were separated by sex, maintained at a density of 200 individuals/cage and provided with ad libitum access to water and sucrose granules until required for testing. Flies were maintained continuously under controlled laboratory conditions before and during experiments: 25 ± 1 °C, 60 ± 10% RH and 12:12 h (light:dark (L:D)) photoperiod. At 24 h prior to experiments, the sucrose was removed from the cage to induce hunger whereas the water source remained available. Adult flies were between 5 and 25 days old when used in experiments—that is their first quarter of their life (*A. ludens* and *A. obliqua* adults usually live for approximately 90 days under laboratory conditions) [22].

A laboratory colony of *D. suzukii* was started in the Instituto de Ecología AC, Xalapa, Veracruz, using adults obtained from naturally-infested wild blackberry, *Rubus fruticosus* L., collected at Xico, Veracruz (19 25′59″ N; 97 1′58″ W, 1385 m altitude). Adults were allowed to oviposit in a cornmeal-based artificial diet [21] that was previously dispensed into 300 mL plastic cups and covered with a fine nylon gauze. Flies were reared and all experiments were performed under controlled laboratory conditions: 24 ± 1 °C, 60 ± 10% RH and 12:12 h (L:D) photoperiod. To control age, females and males were collected every day from emergence and kept together in cages until required for experiments. Water and food (sucrose + yeast) were provided to adults ad libitum [23,24]. To induce hunger, flies were starved for 12 h prior to experiments but water was continuously available.

Propylene glycol (PG) (1,2-propanediol; C_3_H_8_O_2_) 99.9% purity (J. T. Baker, Baker S.A. de C.V., Xalostoc, Estado de México, Mexico), glycerol (1,2,3-propanetriol; C_3_H_8_O_3_) 99.5% purity (Farmacia ABS S. de R.L., Xalapa, Veracruz, Mexico) and sucrose (J. T. Baker, Baker S.A. de C.V., Xalostoc, Estado de México, Mexico) were obtained from commercial suppliers. The sweetener erythritol ((2S,3R)-butane-1,2,3,4-tetrol; C_4_H_10_O_4_) was purchased from Baolingbao Biology Company Ltd., Yucheng, Shandong, China.

### 2.2. Concentration–Mortality Response and Regurgitation of Polyols in A. ludens

Each individual fly was placed in a 300 mL clear plastic cup which was sealed using a nylon gauze lid. Half of the cups contained a male and the other half containted a female fly (1:1 ratio). PG or glycerol were mixed with water at 20%, 33% and 50%. Sucrose (50% *w*/*v*) and green food dye (Deiman S.A. de C.V, Mexico) (0.5% *v*/*v*) were mixed with the polyol solutions to stimulate consumption and to confirm the ingestion of the polyol solution by observation of the color of each fly’s gut through its abdomen. After adding sucrose and dye, concentrations of glycerol and PG offered to the flies were equivalent to 1.67 M, 2.78 M and 4.18 M. As a control, we used 50% sucrose solution equivalent to a concentration of 1.8 M. A 1 mL volume of each polyol + sucrose mixture was placed on a 2.5 mL plastic cap with a piece of sterile gauze on the bottom to facilitate feeding and reduce the risk of insect drowning. The plastic cap was placed at the bottom of each cup. After 24 h, the plastic cap was removed and flies were offered a new plastic cap containing 50% sucrose solution. A total of 80 flies (40 males and 40 females) were assigned to each treatment. Flies were incubated under controlled laboratory conditions and checked on a daily basis. Dead flies and the number of colored regurgitated droplets on the sides and base of each cup were counted daily for three days after treatment. Tephritids usually deposit lines of individual droplets of regurgitate onto the resting substrate for later re-ingestion [25]. Flies were carefully transferred to clean cups every day to avoid counting drops more than once.

### 2.3. Effect of Glycerol and PG on Body Weight in A. ludens and A. obliqua

To determine whether polyol-induced regurgitation affected the body weight of treated flies, 4.18 M glycerol and 4.18 M PG treatments were mixed with 50% sucrose solution and were then offered to flies. Control flies were offered 50% sucrose solution alone. Solutions were offered to a total of 189 individuals of each treatment for *A. ludens* and 134 individuals per treatment for *A. obliqua* over a 24 h period, as described in the previous experiment. Flies were individually weighed on three occasions: (1) immediately prior to the 24 h starvation period, (2) at the end of the 24 h starvation period prior to treatment and, (3) at 24 h after the end of the polyol ingestion period. Only flies that had ingested the polyol treatment (indicated by a green-colored abdomen) were weighed on the third occasion. Flies that did not consume experimental solutions were eliminated from the experiment and their weights taken at earlier time points were removed from the dataset. Each individual fly was placed in a pre-weighed cup and weighed to an accuracy of 0.1 mg using an analytical balance (Sartorius CPA324S, Goettingen, Germany). The number of dead flies and number of regurgitated drops were recorded at 24 h post-treatment (after the end of the polyol ingestion period) following the steps described in the previous experiment (Section 2.2).

### 2.4. Effect of Glycerol and PG on Mortality and Regurgitation of D. suzukii

Groups of three-day-old *D. suzukii* adults of both sexes were starved for 12 h (access to water was available) and were then randomly assigned to 4.18 M glycerol or 4.18 M PG both mixed with 50% sucrose, or a 50% sucrose control treatment. Each treatment was provided on a plastic cap on the bottom of a 300 mL plastic cup for a 24 h period. A total of 120 cups were set up for each treatment. Each cup contained a single fly (replicate). After 24 h of exposure, the polyol treatment was removed and a 50% sucrose solution was provided, as described in the first experiment. Dead flies and the number of regurgitated food drops were counted and recorded at 24 h after the end of the period of polyol consumption as most mortality and regurgitation was observed in the 24 h period post-treatment.

### 2.5. Proboscis Extension Reflex to PG, Glycerol and Sucrose

This experiment tested whether flies exhibited the proboscis extension reflex (PER) to various concentrations of polyols compared to a sucrose or water control [26]. As in the previous experiments, *Anastrepha* flies were starved for 24 h prior to use, whereas *D. suzukii* flies were starved for 12 h prior to use. Each fly was tested once with one of the following six liquids: (1) 4.18 M PG in 50% sucrose solution, (2) 4.18 M glycerol in 50% sucrose solution, (3) 100% PG, (4) 100% glycerol, (5) 50% sucrose solution, and (6) water alone. Flies were never tested against more than one treatment. Each solution was presented to an individual fly using a calibrated glass micropipette (5 µL capacity). The flies were 10–13 days old in the case of *Anastrepha* spp. and 3–5 days old in the case of *D. suzukii*. All flies had access to granular sucrose and water ad libitum in cages before the starvation period. To fix the fly, water-soluble acrylic paint (Vinci, Dixon S.A. de C.V., Tultitlán, Mexico), was used to glue the tip of a toothpick to the fly’s scutellum. Holding the fly by the toothpick, the end of the capillary tube was placed close to the fly’s head until the taste receptors on the front legs (tarsi) of the insect touched the tube. The number of times the fly extended its proboscis during a 10 second period was recorded. This procedure was performed five times with each fly for just one of the treatments. Flies were allowed to rest for 10 seconds following each exposure to the test solution. The total number of proboscis extensions in the five periods was considered as the response variable. Depending on availability, different numbers of flies were used for each species and each treatment (*A. ludens n* = 40, *A. obliqua n* = 26, *D. suzukii n* = 10 flies per treatment, respectively).

As optical refractivity is often used to characterize the concentration of sugars in solutions, we wondered whether refractivity index values could be used as an indicator of polyol attractiveness and regurgitation properties. This is because the refractive properties of polyols differ according to their structure [27]. To examine this idea we measured the refractive index (Brix) of 4.18 M solutions of PG and glycerol and pure preparations using a portable refractometer (USA Refracto 30PX, Mettler-Toledo, Columbus, USA). Water and 50% sucrose solution were included as reference substances.

### 2.6. The Effect of Erythritol on Regurgitation and Mortality in A. ludens

As erythritol had previously been reported to be insecticidal in Diptera, the effect of erythritol on regurgitation and mortality was examined in *A. ludens* using three concentrations that had been tested previously in other species: 2.78 M, 1.78 M and 1.0 M erythritol. These solutions were prepared in 50% sucrose solution, as described in our previous studies, to increase palatability to *A. ludens*. Control insects were fed on 50% sucrose alone. Each treatment was offered to 30 individual flies, 15 of each sex. We followed the methods used in the first experiment considering mortality and regurgitated droplets as the response variables. Dead flies and regurgitated droplets were counted daily during the three days following the 24 h treatment period. Flies that did not die in the three-day post-treatment period were considered for survival analysis.

### 2.7. Statistical Analyses

The numbers of regurgitated droplets were analyzed using a generalized linear model (GLM) with Poisson errors, a log-link function. The prevalence of mortality was analyzed by fitting GLMs with a binomial error distribution specified and a log-link function [28,29]. Contrasts were used to test for differences in levels within a variable. For continuous response variables such as fly-weight, analysis of variance (ANOVA) and type III significance tests that remove the order dependency in the model were used with mean separation by Tukey test (JMP v. 9, SAS Institute, Cary, USA). Regurgitation droplet data was subjected to a non-parametric Kruskal–Wallis test with treatment or sex as the explanatory factor and the Dwass–Steel–Critchlow–Fligner (DSCF) multiple comparison procedure in the R-based Jamovi package [30]. Differences in mortality among treatments were examined by log-rank survival analysis using the Jamovi death watch module.

## 3. Results

### 3.1. Concentration–Mortality Response of Polyols in A. ludens

**Figure 1:**
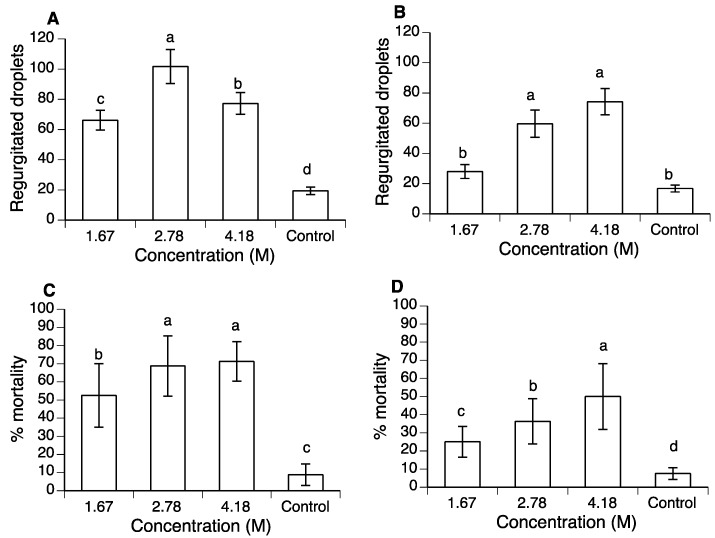
(**A**) and (**B**) Mean (± standard error (SE)) number of regurgitated droplets deposited on cup walls by *A. ludens* flies exposed to three different concentrations of (**A**) propylene glycol (PG) and (**B**) glycerol in mixtures with sucrose. (**C**) and (**D**) Percentage of mortality observed at 72 h post-treatment for (**C**) PG and (**D**) glycerol treatments, respectively. Columns headed by the same letter are not significantly different (generalized linear model (GLM) post-hoc contrasts, at the *p* = 0.05 level).

Consumption of polyols resulted in a significant increase in regurgitation for PG (χ^2^ = 89.65, d.f. = 3, *p* < 0.0001) and glycerol-treated flies (χ^2^ = 59.00, d.f. = 3, *p* < 0.0001) compared to the control treatment. Normal regurgitation behavior of *A. ludens* involved an average of 16–20 droplets per fly during the three-day study (Figure 1A,B). Ingestion of polyol solutions resulted in a 2- to 2.5-fold increase in regurgitated droplets in PG-treated flies and was highest in the 2.78 M PG treatment (Figure 1A). Flies that consumed 2.78 M or 4.18 M glycerol also produced approximately twice the number of regurgitated droplets as the control flies, whereas regurgitation in the 1.67 M glycerol treatment was similar to that of the control (Figure 1B).

Polyol treatment resulted in a marked increase in mortality at 72 h post-treatment in both PG (χ^2^ = 98.98, d.f. = 3, *p* < 0.0001) and glycerol-treated flies (χ^2^ = 49.91, d.f. = 3, *p* < 0.0001). At 72 h post-treatment, mortality in the control treatment was less than 10% whereas mortality varied between 53% and 71% in PG treatments (Figure 1C), compared to 22–50% mortality in glycerol treatments (Figure 1D), depending on polyol concentration. Based on these results, the following experiments focused on the use of 4.18 M polyol solutions.

### 3.2. Effect of Glycerol and PG on Body Weight in A. ludens and A. obliqua

**Figure 2:**
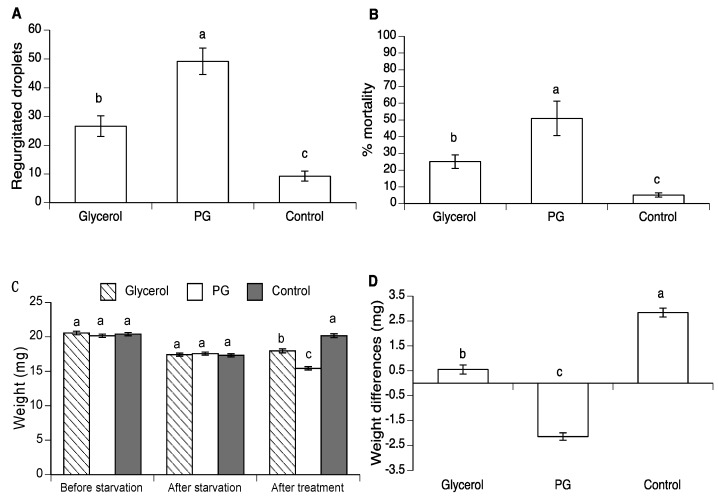
(**A**) Mean (± SE) number of regurgitated droplets deposited on cup walls by *A. ludens* exposed to 4.18 M PG or 4.18 M glycerol in mixtures with sucrose. (**B**) Percentage of mortality observed at 72 h post-treatment. (**C**) Mean (± SE) body weight of flies before starvation, prior to treatment (after starvation), and after treatment. (**D**) Mean (± SE) difference in body weight at 24 h after treatment. Columns headed by the same letter are not significantly different (GLM post-hoc contrasts, at the *p* = 0.05 level).

As observed in the previous experiment, the average number of regurgitated droplets at 24 h post-treatment was approximately two and five times higher in the glycerol and PG treatments respectively, compared to the control (χ^2^ = 72.49, d.f. = 3, *p* < 0.0001) (Figure 2A). Similarly, mortality was significantly higher in the polyol treatments (25–48%) compared to 5.5% in the control (χ^2^ = 103.85, d.f. = 3, *p* < 0.0001) (Figure 2B). Live fly weight did not differ significantly prior to starvation (F_2,548_ = 0.72, *p* = 0.48), or immediately prior to treatment (F_2,548_ = 0.23, *p* = 0.79) (Figure 2C). In contrast, at 24 h post-treatment the average weight of PG-treated flies and glycerol treated flies was significantly reduced compared to flies in other treatments (F_2,548_ = 76.43, *p* < 0.0001). Comparison of weight after starvation and final weight indicate that PG fed flies lost significantly more weight than flies of other treatments whereas the weight loss in glycerol-treated flies was intermediate (F_2,548_ = 120.04, *p* < 0.0001) (Figure 2D).

**Figure 3:**
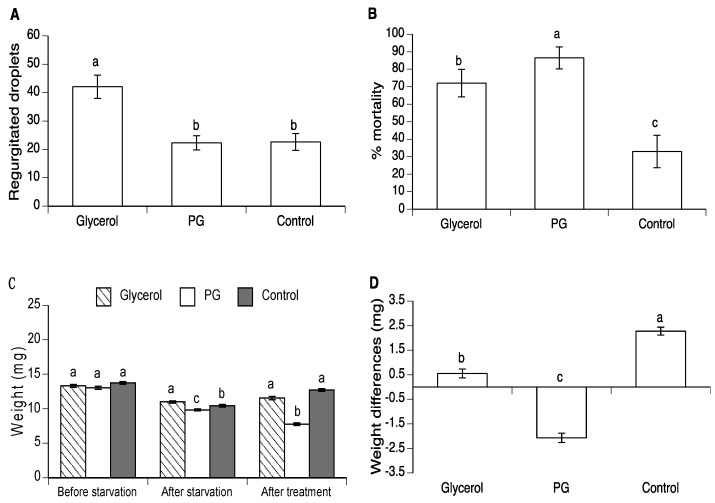
(**A**) Mean (± SE) regurgitated droplets deposited on cup walls by *A. obliqua* exposed to 4.18 M PG or 4.18 M glycerol in mixtures with sucrose. (**B**) Percentage of mortality. (**C**) Mean (± SE) fly body weight before starvation, after starvation (prior to treatment) and at 24 h after treatment. (**D**) Mean (± SE) difference in body weight before treatment and at 24 h post-treatment. Columns headed by the same letter are not significantly different (GLM post-hoc contrasts, at the *p* = 0.05 level).

For *A. obliqua*, the average number of regurgitated droplets was highest in the glycerol treatment, whereas the PG treatment was similar to that of the control (χ^2^ = 24.10, d.f. = 2, *p* < 0.0001) (Figure 3A). In contrast, mortality at 24 h post-treatment was highest in PG-treated flies, lowest in the control and intermediate in the glycerol treatment (χ^2^ = 122.45, d.f. = 2, *p* < 0.0001) (Figure 3B). The initial weight of flies did not differ significantly among treatments (F_2,392_ = 2.90, *p* = 0.0533). However, immediately prior to treatment, fly weight differed slightly but significantly (F_2,392_ = 12.7, *p* < 0.0001). At 24 h post-treatment, fly weight differed markedly (F_2,392_ = 164.55, *p* < 0.0001) (Figure 3C). As a result, the mean change in body weight was strongly negative in PG treated flies (mean weight at 24 h post-treatment: 7.75 ± 0.18 mg), strongly positive in control insects (12.71 ± 0.19 mg), and intermediate in the glycerol treatment (F_2,392_ = 150.98, *p* < 0.0001) (Figure 3D).

### 3.3. Effect of Glycerol and PG on Mortality and Regurgitation of Drosophila suzukii

**Figure 4:**
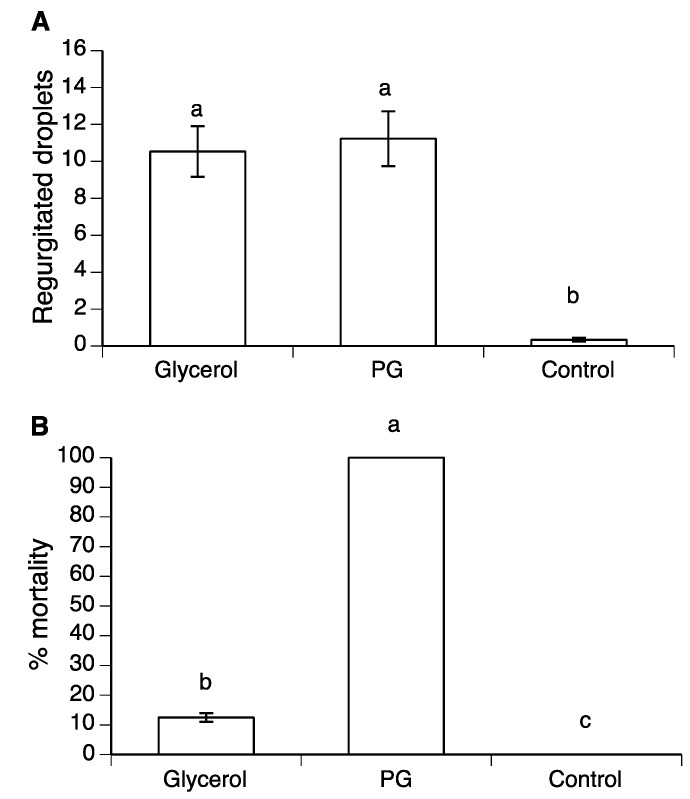
(**A**) Mean (± SE) regurgitated droplets deposited on cup walls by *D. suzukii* flies exposed to 4.18 M PG and 4.18 M glycerol solutions. (**B**) Percentage of dead flies at 72 h after treatment with 50% concentrations of PG and glycerol. Control flies consumed 50% sucrose solution. Columns headed by the same letter are not significantly different (GLM post-hoc contrasts, at the *p* = 0.05 level).

Regurgitation behavior in *D. suzukii* was uncommon in the control group and averaged less than 0.5 droplets/fly/day over the three-day observation period (Figure 4A). In contrast, both glycerol and PG treated flies exhibited frequent regurgitation with an average of >10 droplets/fly/day (χ^2^ = 111.8, d.f. = 2, *p* < 0.0001). No mortality was observed in control flies during the post-treatment period and only a low prevalence of mortality occurred in the glycerol treatment (Figure 4B), whereas 100% of the PG-treated flies died within the three-day post-treatment period (χ^2^ = 382.8, d.f. = 2, *p* < 0.0001).

### 3.4. Proboscis Extension Reflex Studies

**Figure 5:**
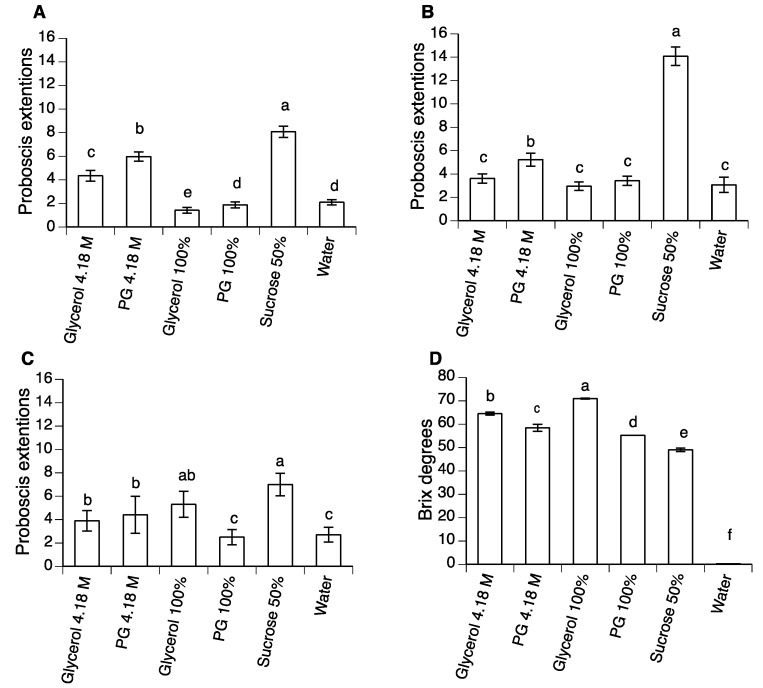
Mean (± SE) number of proboscis extension responses observed by adults of (**A**) *A. ludens*, (**B**) *A. obliqua*, and (**C**) *D. suzukii* following five exposures to 4.18 M or 100% PG or glycerol. (**D**) Refractivity readings (Brix) of 4.18 M or 100% PG or glycerol. Columns headed by identical letters are not significantly different (contrast for GLM, or Tukey test for ANOVA at *p* > 0.05).

The proboscis extension reflex (PER) was examined during exposure to six treatments: 4.18 M and 100% PG, 4.18 M and 100% glycerol, and 50% sucrose or water (controls). Both species of *Anastrepha* presented their highest response to 50% sucrose treatment (*A. ludens*, χ^2^ = 277.40, = 5, *p* < 0.0001; *A. obliqua*, χ^2^ = 232.64, d.f. = 5, *p* < 0.0001) (Figure 5A,B), whereas for *D. suzukii*, the response to 50% sucrose was similar to that for 100% glycerol, but lower in all other treatments (χ^2^ = 22.02, d.f. = 5, *p* < 0.001) (Figure 5C). In most cases, 4.18 M PG also elicited a strong PER response in all species tested.

The refractivity of polyols and polyol solutions varied significantly (F_5,24_ = 1159.6, *p* < 0.0001), but this parameter was not a useful indicator of likely regurgitation response or insecticidal activity in any of the species tested (Figure 5D).

### 3.5. The Effect of Erythritol on Regurgitation and Mortality in A. ludens

**Figure 6:**
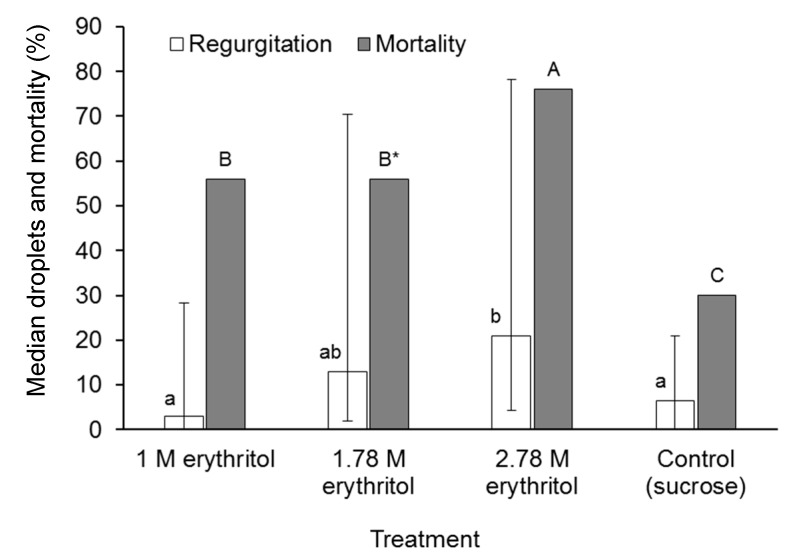
Median regurgitated droplets (open columns) deposited on cup walls by *A. ludens* flies individually exposed to three different concentrations of erythritol in 50% sucrose or 50% sucrose alone (control), and percentage of mortality (shaded columns) of flies in the three-day post-treatment period. Vertical bars indicate interquartile range. Identical letters above columns indicate no significant differences in regurgitation (lower case, Dwass–Steel–Critchlow–Fligner (DSCF) multiple comparison test *p* > 0.05) or mortality (upper case, log-rank test, *p* > 0.05). * Comparison of 1.78 M erythritol treatment and control was borderline significant (log-rank test, *p* = 0.055).

When erythritol was presented in mixtures with 50% sucrose, all of the flies consumed the polyol–sucrose mixtures during the treatment period (as evidenced by the food dye-induced coloration of their gut). Regurgitation of droplets increased significantly from a median of three droplets/fly (mean ± SE: 18.5 ± 5.3) in the 1 M erythritol treatment, which was similar to the control (mean 17.1 ± 4.3), to a median of 21 droplets/fly (mean 43.0 ± 8.2) in the 2.78 M erythritol treatment (Kruskal–Wallis H = 8.11, d.f. = 3, *p* = 0.044) (Figure 6). Regurgitation did not differ significantly by sex (Kruskal–Wallis H = 0.02, d.f. = 1, *p* = 0.876). Fly mortality was higher than the control (30% mortality) in all erythritol treatments, but was highest in the 2.78 M erythritol treatment, in which 76% of flies died in the three day post-treatment period (Figure 6). In the case of the 1.78 M erythritol treatment, the difference with the control was borderline significant (log-rank test z = −1.922, *p* = 0.055).

## 4. Discussion

In this study, we tested the effect of two polyols, glycerol and propylene glycol (PG), on three dipteran species that are major agricultural pests: two tephritids of the genus *Anastrepha* and the invasive drosophilid *D. suzukii*. When administered in mixtures with 50% sucrose, both polyols (particularly PG) caused elevated incidence of regurgitation in most of the species tested and an increased prevalence of mortality at 24–72 h post-treatment. The association between regurgitation and mortality in polyol-treated flies was corroborated using erythritol, a polyol with known insecticidal properties.

Ingestion of 2.78–4.18 M PG over a 24 h period killed a high percentage of adults of the dipteran species tested, generally in a concentration dependent manner, whereas glycerol had a lower insecticidal effect in *Anastrepha* spp. and little insecticidal effect in *D. suzukii*. In terms of mortality, PG was most toxic to *D. suzukii*, of intermediate toxicity to *A. obliqua* and least toxic to *A. ludens*.

Previous studies on the toxicity of polyols in Diptera have focused on erythritol and other non-nutritive sweeteners such as mannitol, xylitol and sorbitol that have lower insecticidal activity than erythritol. These studies consistently report dose-dependent mortality in drosophilids [10,11,31,32], a muscid [26], and the tephritid *Bactrocera dorsalis* [13] at 3–7 d following ingestion of erythritol at concentrations usually between 0.1 M and 2 M, compared to the 1.0–2.78 M range that we employed in the present study. These studies generally involved ingestion of erythritol alone or in combination with sucrose during the entire duration of the experiment, which contrasts with the shorter 24 h period of ingestion of the polyol treatments that we employed.

The mechanism by which erythritol exerts its insecticidal effect is unclear but does not appear to involve starvation due to reduced feeding responses [32], or the non-nutritive characteristics of this compound [10,11], or its capacity to inhibit the uptake of nutritive sugars in the gut [26,33]. Rather, it is has been suspected that erythritol consumption induces an osmotic shock that the insect cannot control, resulting in physiological trauma and death. Indeed, in choice tests, *D. suzukii* that consumed erythritol also consumed an elevated volume of water, suggesting that flies were attempting to compensate for the extreme osmotic pressure resulting from high levels of erythritol in the hemolymph [11,34]. The present study reveals that hyper-regurgitation is also a clear response to erythritol ingestion that may contribute to insect mortality in this and several other insecticidal polyols.

The relationship between the physiological effects of PG and glycerol and insect mortality have not been studied previously. Although both glycerol and PG are considered to be safe for humans [20,35,36], the reported effects of PG toxicity in humans include hyperosmolarity, lactic acidosis, and acute kidney injury [37] and a high intake of glycerol can produce similar symptoms in rats [16,38]. The osmotic properties of polyols may therefore impact the internal osmotic conditions of insects so rapidly and to such a degree that it is physiologically impossible to compensate for their effects, resulting in death.

In the present study we identified an additional mechanism through which polyols exert insecticidal effects, that of lethal regurgitation. Both PG and glycerol induced regurgitation in *A. ludens*, *A. obliqua* and *D. suzukii*. This resulted in a marked reduction in body weight compared to sucrose-fed controls, presumably due to continuous regurgitation of fluids from the crop resulting in acute dehydration and death within 24–72 h. In a previous study, a regurgitation response was observed following ingestion of erythritol by the house fly *Musca domestica* L., but this was not quantified and was not differentiated from defecation [12]. In our study we demonstrated that erythritol exacerbated regurgitation, as did PG and glycerol. However, this substance did not kill flies as fast and in the same numbers as PG. It seems that regurgitation is only part of the negative effects that polyols provoke in flies. Fly mortality may well represent a consequence of the hygroscopic nature of PG, that could dehydrate the insect’s body [39].

Emesis or vomiting of toxic compounds or pathogens ingested during feeding serves to protect individuals from poisoning or infection and is a common response in many vertebrates [38]. In contrast, in insects, regurgitation is part of the digestive process or a mechanism to share food with conspecifics or offspring [40,41]. Regurgitation is also used by some insects to alter the energetic density of their food [25,42,43] or is employed as a defensive response to avoid predation [44].

PER experiments demonstrated that compared to 50% sucrose, PG and glycerol did not elicit strong PER responses in *A. ludens* or *A. obliqua*, whereas a slightly stronger response was observed in *D. suzukii* (Figure 5A–C), possibly because low levels of glycerol are naturally present in the diet of this insect [45]. Similar studies on *D. melanogaster* revealed reduced PER responses to glycerol compared to sucrose or a range of other sugars, and very low PER responses to sorbitol when sensilla on the foreleg (tarsi) or labellum were stimulated [46,47]. Although the PER responses to PG and glycerol were lower than to 50% sucrose, the fact that all species were observed to feed on polyol solutions (as evidenced by the green colored abdomens of experimental insects) indicates that they were not repellent and were consumed during each of the experiments that we performed. The presence of sucrose in the PG and glycerol treatments we tested is likely to have improved polyol consumption by flies given that sucrose elicits a strong feeding response in all the species tested [48,49,50,51]. This is an important characteristic that could favor the use of polyols mixed with sugar baits. For example, mixing them with attractive toxic sugar baits, developed to “attract and kill” mosquitoes, may result in an almost harmless combination that could be used as an adulticide inside houses [52,53]. Additionally, by delaying evaporation, PG can be combined with devices that allow an increase the time the mixture could be active in the field [54]. Moreover, PG does not have negative effects on human respiratory function or produce ocular irritation; thus, spraying could be another method of application [40]. Despite these desirable characteristics, further studies that include the effect of polyols on bait attractiveness must be performed to ensure their efficacy in pest control.

## 5. Conclusions

In conclusion, our findings indicate that PG induced up to a five-fold increase in the incidence of regurgitation in *A. ludens* and more than 20-fold increase in regurgitation in *D. suzukii*. This may be a response to the osmotic shock suffered by insects that consumed these substances, or an unusual example of emesis as a means to purge a noxious compound from affected individuals. However, *A. obliqua* suffered high mortality in the absence of marked regurgitation following ingestion of PG. Similarly, *D. suzukii* adults that consumed glycerol regurgitated as frequently as PG-treated insects but experienced a low prevalence of mortality compared to PG-treated individuals (Figure 4A,B). Erythritol in sucrose solution also elicited a significant regurgitation response and elevated mortality in *A. ludens*. It seems, therefore, that extreme levels of regurgitation could be a previously unrecognized mechanism by which polyol-treated flies become lethally dehydrated. The importance of this mechanism may vary with species, as observed in the present study, but the use of polyol compounds in combination with sucrose in fly baits might contribute to the efficacy of dipteran pest control measures that are innocuous to humans and other vertebrates. The safety of polyol-based baits to beneficial insects such as insect pollinators is an issue that requires additional study, given that the sweet taste of these compounds may make them attractive to non-target invertebrates.
